# Youth and healthcare workers’ perspectives on the feasibility and acceptability of self-testing for HIV, Hepatitis and Syphilis among young people: Qualitative findings from a pilot study in Gaborone, Botswana

**DOI:** 10.1371/journal.pone.0288971

**Published:** 2023-07-20

**Authors:** Kyegombe Nambusi, Ajibola Gbolahan, Sakoi-Mosetlhi Maureen, Rebatenne T. Tsholofelo, Anderson M. Motswedi, Gaseitsiwe Simani, Makhema Joseph, Ngwenya Una, Moyo S. Sikhulile, Sauzet Odile, Mupfumi Lucy

**Affiliations:** 1 Department of Global Health and Development, London School of Hygiene and Tropical Medicine, London, United Kingdom; 2 Botswana Harvard AIDS Institute Partnership, Gaborone, Botswana; 3 Department of Immunology & Infectious Diseases, Harvard T.H. Chan School of Public Health, Boston, MA, United States of America; 4 Botswana Family Welfare Association, Gaborone, Botswana; 5 Department of Epidemiology and International Public Health, Bielefeld School of Public Health (BiSPH), Bielefeld University, Bielefeld, Germany; Stellenbosch University, SOUTH AFRICA

## Abstract

**Introduction:**

Little is known regarding the attitudes and perspectives of young people and healthcare workers in Botswana about dual self-testing for HIV and STIs including its acceptability, or their perceptions of the opportunities and limitations of this approach.

**Methods:**

From July to November 2021, 25 young people and 6 healthcare workers were purposively sampled for in-depth telephone interviews conducted in English or Setswana. The interviews followed a semi-structured topic guide, were audio recorded, transcribed, and analysed thematically using deviant case and constant comparative techniques. The study was part of a pilot project evaluating dual self-testing for HIV and STIs among young people in Gaborone.

**Results:**

We found that most of the young people were already aware of their HIV status and were motivated to participate in self-testing mainly because they were interested in learning their STI status. Whilst most were excited about the autonomy and convenience offered by self-testing, some participants expressed nervousness particularly of the finger-prick process, and preferred healthcare worker-administered tests. Both young people and healthcare workers raised concerns about the potential negative mental health outcomes of unexpected test results and emphasized the importance of pre- and post-test counselling and seamless linkage to care.

**Conclusion:**

Dual self-testing for HIV and STIs has the potential to empower young people to take control of their sexual health. However, it is crucial to ensure that proper support and counselling services are in place, along with effective mechanisms for linkage to care. This study emphasizes the importance of integrating pre- and post-test counselling into self-testing programs to ensure that young people feel adequately supported throughout the testing process. By doing so, self-testing can become a valuable tool for improving the sexual health outcomes of young people in Botswana.

## Introduction

Young people aged 16–24 years continue to face considerable structural and physical barriers in obtaining sexual and reproductive health information and services [1] leading to the increases in the rates of sexually transmitted infections (STIs) such as syphilis [2], and HIV [3]. Globally, 40% of all new HIV cases in persons of reproductive age occur among young people, yet less than half know their HIV status [3,4]. Although Botswana has made tremendous progress towards meeting the UNAIDS 95-95-95 goals, gaps remain in reaching men and young people [5,6]. Young people in Botswana account for three in every ten new HIV infections [7] but incomplete data disaggregation makes it difficult to fully understand the magnitude of the HIV and STI epidemic in this group.

Whilst the COVID-19 pandemic caused disruptions to testing for HIV, syphilis and hepatitis reported in most countries including Botswana [8], it also led to an unprecedented shift to community delivery of treatment, use of telemedicine and an increase in HIV self-testing [9]. The potential of self-testing for HIV, Hepatitis and STIs as a central strategy to a self-care model for sexual reproductive health (SRH) among young people in Botswana is currently unknown, and views on self-testing among youth populations in other contexts are mixed [9–13]. Whilst confidentiality of HIV test results, convenience, and perceived autonomy and self-empowerment are cited by most young people as benefits of self-testing [13], some young people preferred healthcare provider testing, voicing concerns about lack of privacy to conduct self-testing at home and fear and anxiety of test results in the absence of face-to-face counselling [11].

Provision of youth friendly health services has been shown to provide high levels of satisfaction among young people in Botswana [14], who describe the attitude of health care providers as a significant barrier to the access of SRH services [15]. Through an observational pilot study, we sought to determine the feasibility and acceptability of self-testing for HIV and STIs among young people aged 18–24 years in Botswana within the context of a youth-friendly clinical environment. Our central hypothesis was that providing young people agency over their testing and access to care would address the existing structural barriers to SRH care faced by young people. To our knowledge, this is the first description of dual self-testing for HIV and STIs in this population in Botswana.

## Methods

### Study context

Botswana is an upper middle-income country. While there is some socio-economic variability within Gaborone it’s capital, access to basic health care is free at the point of delivery for all. The availability of more advanced services, including more specialised SRH testing and diagnostic services, varies by location. All residents of Gaborone are intended to be no more than five kilometres from a health facility. Owing to stigma, young adults are however known to travel away from their areas of residence to access SRH services. The cost of education up to the end of secondary school is free for all in government-supported schools and supported through a grant scheme at tertiary level.

### Sampling and data collection

Participants were recruited into the study between 30 July and 30 November 2021 at the Botswana Harvard Partnership (BHP) clinical research sites in Princess Marina Hospital, Gaborone and at the youth friendly clinic of the Botswana Family Welfare Association (BOFWA) in Gaborone. A total of 200 young people (female and male) aged 18–24 years, citizens of Botswana, and able to provide independent consent for study participation, were invited to participate in the *Ichecke*! study. During the initial study visit their eligibility was assessed, informed consent for all study procedures was obtained, and pre-test counselling delivered by a trained study research assistant. The research assistant then provided training on self-testing along with a pictorial usage instruction sheet in English and Setswana before participants used the Accu-Tell rapid combo test kit for Hepatitis B surface antigen (HBsAg)/Hepatitis C virus (HCV)/HIV/Syphilis [16] (a finger prick test) in a private booth/room at the study research clinic. The research assistant then provided post-test counselling to the study participant. Blood was also drawn for confirmatory laboratory testing for the diagnostic accuracy evaluation. Participants were assigned a study ID and any identifiable data such as consent or locator forms was kept in a locked cabinet accessible only by the study coordinator and principal investigator. De-identified data were entered into RedCap. All participants received cash compensations for their time and travel costs to the clinical site as approved by the IRBs.

Twenty-five participants were purposively sampled to participate in an in-depth telephone interview which was conducted one to two weeks following the entry visit. Sampling criteria for the in-depth interview were designed to maximise the heterogeneity of the sample to include females and males, different ages, as well as individuals with different education levels. In addition, in-depth interviews were conducted with 6 healthcare workers who were included in the study if they were from the Ministry of Health, SRH services providers at other clinical sites not involved in the study and private providers of SRH services. These selections ensured that the healthcare providers were currently providing sexual and reproductive health which would make their perspectives and experiences relevant to the study.

Before commencing the interviews, and to reconfirm participants’ consent to participate, research assistants reminded them of the discussion that they had during the entry visit, that their participation was voluntary, and that they had the right to end the interview at any point without providing a reason. Healthcare workers provided written informed consent at the time of their interview with a study research assistant. Interviews were conducted in English or local language Setswana using a semi-structured topic guide and were audio recorded.

### Analysis

De-identified data entered RedCap was used for the analysis. Team de-briefs were conducted to discuss the data and reflect on emerging themes. Transcripts of the interviews were also reviewed by the first author during data collection to explore themes for deeper exploration, and for quality monitoring purposes. Completed interviews were transcribed and translated into English (where necessary) for analysis. Data analysis was thematic and complemented by deviant case and constant comparative techniques [17]. Using the topic guides, a review of the literature, and the themes that emerged during the transcript reviews, the first author read the transcripts extensively and then manually organised the data into thematic codes, with these codes used to reduce the data and summarise it by theme. Following intensive reading, the coded data were compared across sex and education level and summarised in the model of data that is presented below.

### Ethical considerations

All participants provided written informed consent to participate in this study. Ethical approval for this study was provided by the ethics committees of the Botswana Ministry of Health and Wellness Health Research Unit (REF: HRDC#00931) and the London School of Hygiene and Tropical Medicine (REF: 25244 /RR/24063).

### Inclusivity in global research

Additional information regarding the ethical, cultural, and scientific considerations specific to inclusivity in global research is included in the (S1 Checklist).

## Results

Between July and November 2021, 200 young people (63% female, median age, 21 years, IQR: 20–23) enrolled into the study evaluating the feasibility, accuracy, and acceptability of dual self-testing for HIV and STIs. All young people tested themselves in the presence of the research assistant and viewed their results within 15 minutes. The research assistant provided pre-and post-test counselling and explained the results. Additionally, the research assistant was trained to identify and manage any distress or negative emotional reactions that participants may experience during or after testing and provide appropriate referrals for further care as needed. Results of the confirmatory laboratory test were available within 5 days and young people were called to the clinic to view their results and for a discussion on the next steps. Twenty-five of the young people were invited to participate in in-depth interviews to understand their experience accessing SRH services as well as their perspectives and attitudes towards self-testing. Fifteen of these (60%) were female and in either college or high school (Table 1). Twenty-three already knew their HIV status and had tested mainly at a health facility, while only 6 had tested at a voluntary counselling centre. There was perfect agreement between the results of the self-test and the laboratory confirmatory tests. The healthcare workers interviewed had vocational experience working either at the Ministry of Health or providing SRH services to young people in the public sector or as key partners in non-governmental organisations focusing on SRH.

**Table 1:** Demographic characteristics of young people taking part in the in-depth interviews.

Variable	He (n = 10)	She (n = 15)
Age (yrs., Median (Q1, Q3)	19 (19, 21)	21 (20, 22)
School status: currently in school	5	10
University/College High School	3 2	9 1
School status: not in school	5	5
Ever faced challenges with accessing healthcare	0	4
l feel comfortable speaking to my healthcare provider about contraceptives	9	15
l feel comfortable speaking with my medical care provider about testing for HIV & STIs	10	15
l feel that my medical care providers judge me when l seek care	1	2
Have you had a sexual encounter	8	13
Age at first sex (yrs., Median (Q1, Q3)	17.5	18
Number of sexual partners one two to three more than 3	1 2 5	4 3 6
How often do you use condoms? never often always	0 3 5	1 9 3
If identify as she, how often do you use contraceptives. never often		12 3
Ever tested for HIV	8	15
Result of previous HIV test Positive Negative	0 8	3 12
Ever tested for STI (e.g., syphilis or gonorrhoea)	1	3
Ever treated for an STI	1	4

### 1. Perceptions of sexual and reproductive health services for youth

Both male and female participants described being aware of places where they could access sexual and reproductive health services including through hospitals, clinics, and their schools, often as part of other health services. Amongst these, some described being happy with the services that they received and not having experienced any challenges in accessing the services. Many, particularly females, however, described healthcare worker-related challenges in accessing these services including perceived judgement, stigmatisation for being sexually active, and being at the ‘mercy’ of healthcare workers to access services, as two 21-year-old female university students describe:

‘*Actually*, *l had a problem at [name of clinic] youth friendly clinic*. *Last time l tested; the woman was like “why are you testing*? *You should test after a year*. *I have to document the main reason why you are testing now and if it’s worth it*, *then l can test you*. *If not*, *l am not going to test you*.*” So*, *you find out sometimes it’s the attitude of the healthcare workers’ [that is challenging]*. *1003 female 21 university*

‘*Most of us when we think of going to the clinic*, *we get scared…most people when they think they’re ill*, *will just say what will other people think when they see me [at the clinic]*? *When l get there what am l going to say*? *Their [healthcare workers] reactions are never ok*, *sometimes they come at you all judgy*. *Maybe you tell them l had unprotected sex*, *and they’re like “Why*, *don’t you know the risks associated with that’*. *1098 female 21 college*.

Healthcare worker-related challenges were also described by the health workers themselves, some of whom concurred with the youth participants that the attitudes of healthcare workers negatively affected some young people’s confidence, willingness, and ability to access sexual and reproductive health services.

‘*In the health facilities*, *we have requested the districts to bring someone for training who is closer to the youth ages…The other hindrance that is there is our attitude as healthcare workers*. *Because you find that even if someone is trained*, *when you see a 13-year-old*, *someone the same age as your daughter*, *coming to access sexual reproductive health services you say*, *“ah have you started already*, *le wena mwanaka (you too my child)”*. *You see such comments stigmatize young people*.*’ HA03 healthcare worker*.

In addition, some of the healthcare workers described youth sexual and reproductive health services to be inadequate because they were concentrated in and around the capital Gaborone, and that they lacked privacy meaning youth sometimes had to queue with other patients, potentially including family members and elderly members of the public in front of whom they would not feel comfortable.

‘*The majority of NGOs*, *for example*, *are only operating in Gaborone*, *the capital city and not in other rural areas where the young people can know about these services and access them*. *Whereas you find that the prevalence of HIV and teenage pregnancy is high in these places [rural areas] because there is a gap when it comes to access*, *there is a gap in awareness*, *there is a gap in knowledge and understanding of how to get what and how’*. *HA02 healthcare worker*.

Both youth participants and healthcare workers further described how the SARS CoVID-2 pandemic had both exposed and exacerbated the challenges faced by youth in accessing sexual and reproductive health services:

‘… *now there is COVID-19 disease; some will say “we are not supposed to go anywhere*, *we are supposed to limit our movement; what if we get to the hospital and we interact with people without knowing if they have the disease or not”*? *A hospital is a place where there are so many patients; you often do not know what someone is at the hospital for*. *It is something that could discourage them from going; because of COVID-19*..*’ 1014 male 19 not in school (high school*, *university*, *or college)*

### 2. Motivations, views, and experiences of self-testing for HIV and STIs

Against the backdrop of challenges in accessing sexual and reproductive health services, youth participants described their motivation for participating in this HIV and STI self-testing study. 92% (23/25) knew their HIV status prior to enrolling in the study. Interestingly, participants’ previous HIV test history did not appear to influence their desire to self-test, as reflected in the response by one of the participants who had not tested for HIV before:

‘*It was my first time to come and test so I thought it would be painful*. *I was also happy because in my life I have always wanted to test myself for different diseases not only HIV*. *I used to get people asking me to go and test for HIV only*, *they used to talk of HIV only*. *When I heard that you could test for other infections*, *I became excited for the opportunity to test for other infections which I have always wanted to test for especially Hepatitis and HIV/AIDS’*. *1014 male 19 not in school (high school*, *university*, *or college)*

Moreover, when asked if they would be interested in testing themselves again, all participants responded they would. The desire to test for sexually transmitted infections other than HIV was also described by some as an opportunity to find out their health status and to seek out treatment for those infections that are treatable, particularly for people who were not confident in the fidelity of their partner:

‘*It’s important for us to test because there is a lot going on*, *(laughs) because we are dealing with unfaithful partners so it’s very very important for us to test occasionally because you don’t know what your partner is doing on the side*. *Not only for HIV/AIDS*, *but for other STIs*. *Because they can be contracted*, *isn’t*? *even if you guys are using protection*, *just to know what’s going on and attend to the problem if it’s there while there is still time’ 1019 female 22 university*.

While many participants described being highly motivated to learn their STI status, a number also described being nervous or fearful of the actual process of pricking their own finger, because they feared the pain and/or injections, were nervous at the sight of blood, or were not confident that they would be able to conduct the test accurately. One participant, however, appreciated the efficiency of the test which meant that through a single finger prick, that they would be able to test for multiple infections:

‘*I enjoyed the fact that it’s an all-in-one device*, *you don’t have to prick many times to draw blood or wait a couple of days to run another test*. *I liked the fact that it was something that l could do*, *and it covered four tests’ 1031 female 21 university*.

### 3. Participants’ views on self-testing for HIV and STIs versus healthcare worker administered testing

Participants’ views on their preference for either healthcare worker-administered or dual self-testing for HIV and STIs, were split. Some participants were very supportive of self-testing for HIV and STIs, especially those who also held less favourable views on youth friendly sexual and reproductive health services. A number of these participants also encouraged wide scale distribution of the self-test kits through schools, clinics, bars and other entertainment areas, tertiary institutions, places where youth congregated such as shopping malls, and in remote and rural areas. They often described the convenience, ability to avoid stigma and judgement, privacy, and empowerment offered by self-testing as beneficial, with the potential to lead to better sexual health outcomes for youth through earlier treatment seeking:

‘*I would encourage young people to use it because one can do it any time*, *at home at their own comfortable time compared to going to the clinic…It is quite beneficial because some people may not feel comfortable being tested by healthcare worker because they fear the healthcare worker might tell someone about their results*. *But doing alone can make you feel free and seek help earlier’ 1007 female 22 university*

*‘I think the self-testing kit is a very good idea for people to know their status. Many people are afraid to go to the testing centres, afraid of being told they have HIV. So, if l have the test kit at home, at least l can test myself then build up courage to go to the clinic to tell them my status*. *I am very positive about this kit.’ 1094 male 19 not in school (high school, university, or college)*

Several healthcare workers also held positive views on youth self-testing for HIV and STIs and concurred with the youth participants that self-testing provided an opportunity to empower young people to be active agents in their own sexual health:

‘*l think it’s [self-testing] long overdue*, *and as a country we came late to the party in terms of acknowledging that self-testing should be one of the initiatives that we do in combatting HIV*, *and even HIV awareness*. *I love self-testing because then it gives the control and responsibility to the person who is doing the self-test on themselves*. *It brings a lot of trust and accountability to people*, *to young people themselves that they are putting matters into their own hands (laughs) when it comes to HIV*. *Which is something that we know was a hinder*, *issues of counselling*, *a young person walking into a clinic and being told all sorts of things*, *because that alone can hinder a young person to actually walk into the clinic and try to test for HIV*. *So*, *l think that self-testing is a good thing*, *with more awareness*, *knowledge and teaching and introducing the concept and initiative to Botswana*, *l think it will be really*, *really good*.*’ HA02 healthcare worker*.

In contrast, other youth participants and some healthcare workers voiced some concerns about self-testing for HIV and STIs for youth for several reasons. First, they described concerns related to how an individual would handle a positive test result if they were on their own and were unsupported:

‘*The potential risks are that some people would not accept their status when they test alone*. *Maybe someone will commit suicide*, *maybe someone will do something silly because they tested alone*, *and they don’t know the next move*, *or they are not confident enough to take the next move when they see their results’*. *1003 female 21 university*

This concern was also voiced by some healthcare workers who noted associations between mental ill health and the risk of HIV and potential negative mental health outcomes for individuals who tested positive and were not sufficiently supported.

Second, one healthcare worker shared their concern that self-testing could lead to youth self-diagnosing themselves and not seeking out medical care, and to increased sexual concurrency if tests were used by prospective partners in advance of sexual activity:

‘*The first one [concern] would be less and less visits to health facility for expert advice*, *because they’d think it’s also a self-diagnosis*. *Secondly l think one other barrier would be uhm*, *how do l put it*. *First it would be self-diagnosis*, *there is probably going to be a higher number of concurrent partners because they’d say*, *“oh we have this self-test kit*, *so let’s test each other*…*oh you’re negative and I’m negative”*. *So*, *it’s going to increase the number of sexual partners that young people are having*, *because now there wouldn’t be that scared that l don’t know your status*. *‘let’s just do a self-test and we will be good” instead of saying “let’s go to the healthcare facility to just have the whole process done for us”‘*. *HA01 healthcare worker*.

Whilst not a widely held view among the healthcare workers interviewed, this healthcare worker was particularly concerned that young people might interpret their results to mean that they are “all clear” of HIV and STIs. Furthermore, they were also concerned that young people could think that with self-testing, that they would never have to seek sexual and reproductive healthcare services from a clinic again in the future, even though the kits do not cover all potential sexually transmitted infections:

‘*So*, *you see this is how l was saying*, *that some of them might think that as long as l have those taken care of*, *l don’t need to know about the other STIs that are not covered by the test*. *So*, *l think the importance of the youth still going to look for help at the healthcare facilities should be emphasized*. *Since the self-test kit does not cover all of the STIs that are a problem with our youth*. *So*, *l think that should be emphasized*, *that regular visits to our healthcare facilities are still important*. *It doesn’t nullify them’*. *HA01 healthcare worker*.

To address these concerns, both youth participants and healthcare workers strongly emphasised the need for good-quality counselling to be provided in conjunction with self-test kits to ensure that users were well prepared, and supported throughout the process of self-testing:

‘..*To self-test right*, *yeah*, *coming from the issue of acceptance and having fear and the like*, *it may be advisable that they self-test in front of a health worker or under supervision of some sort*. *Like maybe after testing*, *you have a health-worker to cross-check the results and see if you need any further evaluation or counselling in regard to the results*.*’ 1031 female 21 university*

‘*Before a person tests*, *they should be counselled that they should accept anything that may happen with the results*, *and they should be able to cope with the situation*’ *1105 female 2*2 *not in school (high school*, *university*, *or college)*

‘*Things such as pre-test counselling should be one of the strong points that we look into*, *to ensure people have the adequate counselling to ensure that no matter how their results are going to be*, *now that they are the ones that will know before anyone else*, *they should know and accept*, *or prepared enough that by the time they get their result they are prepared to accept any result that’s going to come on the test’*. *HA02 healthcare worker*.

‘*[We need] to ensure that pre-test counselling is done prior to someone accesses the self-test kit*. *Ensure that there is a communication channel that allows young people to continue with that process*, *it shouldn’t just be a once-off activity… In order to adopt this initiative*, *we need to ensure that communication channel so that someone can always come back to get those services*. *Also reporting of the result because we know that it is difficult once someone knows they’re HIV positive they won’t tell people*. *So*, *a lot of awareness is needed so people understand what we mean by HIV testing’ HA02 healthcare worker*.

Third, some healthcare workers also emphasised the importance of ensuring that service providers were prepared for and receptive to large increases in demand for sexual and reproductive health services if self-testing became more widespread and accessible. This included having wide availability of confirmatory testing for all the STIs that are included in self-tests which in some clinics would require an expansion of the STI testing facilities that are currently available. It would also require that treatment is widely available to ensure that individuals are able to readily access treatment. Monitoring the uptake and experiences of youth with HIV and STI self-test was also described to be important to monitor equity in access and to assess whether self-testing enabled a broadening of sexual and reproductive health services to youth:

‘*We need to make sure we are checking in with the young people to see if they are being helped once we roll out testing*, *figuring out the profile of young people that it is helping*, *making sure that we are not missing young people out of school or kids that are not very literate*. *So*, *things like that*, *like how do we make sure that we are in touch with all those other young people*. *And*, *getting that feedback about how it’s working for them*. *I think that that’s key*. *Because if we find out the only people using self-testing are the people who are already using the clinic services i*.*e*., *it’s not extending the reach of HIV then it’s not very useful*. *But if we find it’s reaching broader*, *then how do we make sure it continues to do that and that it serves them the way It should*?*’ HA06 healthcare worker*.

## Discussion

This paper draws on qualitative data from a mixed-methods pilot study on the feasibility and acceptability of self-testing for HIV and STIs among young people and healthcare workers in Gaborone. The young people in this study preferred the autonomy and agency that dual self-testing for HIV and STIs offered them. At the same time, they also wanted access to pre-and post-test counselling, a sentiment echoed by the health care workers that were interviewed. Our results also highlight the challenges that young people often face in accessing sexual and reproductive health services including lack of privacy, perceived judgement and negative attitudes of service providers, inconvenience and inaccessibility, and concerns about confidentiality, challenges that healthcare workers in our sample also recognised.

Our findings suggest that while young people may be aware of their HIV status, the majority had never tested for STIs because STI testing is less widely available and may be less highly prioritised in sexual and reproductive health service provision. In Botswana, testing for STIs is not routinely done and management is mainly syndromic. Dual testing for HIV and STIs offers potential avenues for engaging with young people, especially boys, on their sexual health if self-testing for HIV and STIs is presented as an opportunity for them to obtain a fuller understanding of their health [18]. Indeed, this opportunity was described by youth participants in this study to be their main motivation to participate in this study, particularly for those who recognised that it also presented an opportunity to obtain early treatment of potential infections given concerns about the fidelity of their sexual partner.

Furthermore, as noted in other contexts, convenience, autonomy, privacy, ability to avoid stigma and judgement, and confidentiality were also described by participants as further facilitators of self-testing [19]. Several healthcare workers described positive views on youth-self testing for HIV and STIs on the basis that it provided an opportunity to empower young people to be active agents in their own sexual health. Expanded access to STI testing will however require investment in service capacity for confirmatory testing and linkage to care which has implications on service provision and financing.

While many young people described being highly motivated to learn their HIV/STI status, a number also described being nervous or fearful of the actual process of pricking their own finger, because they feared the pain and/or injections, were nervous at the sight of blood, or were not confident that they would be able to conduct the test accurately. Young people’s lack of confidence to properly conduct self-tests and thus preference for healthcare worker-administered tests has also been reported in other contexts [12,19]. A few youth participants and healthcare workers also voiced concerns about whether an individual would be able to handle a positive test result on their own if they were unsupported, with some citing potential negative outcomes include mental health crises and even suicide.

The need for effective pre- and post-test counselling and seamless linkage to care was emphasised by many participants and healthcare workers as an important requirement in the delivery of any self-testing services to mitigate these risks. This finding emphasizes that young people want the agency and convenience of self-testing coupled with safe, and supportive spaces should they choose to engage with the health facilities [12]. Healthcare workers in this study also voiced concerns that HIV and STI self-testing may increase sexual concurrency among young people again highlighting the need for good quality counselling. This study provided pre-and post-test counselling within the health facility where young people conducted the self-test in front of the research assistant. However, we propose an ideal self-care model that would enable young people to access self-test kits from a variety of locations such as schools, malls, bars, sports facilities, and health facilities. Such a model would also include access to an app or web-based application that would enable young people to access peer counsellors or be linked to a healthcare worker as they prefer. This approach would provide a convenient and accessible means for young people to take control of their sexual health through self-testing while also having access to the necessary support and counselling.

### Study limitations

The results should be interpreted considering the limitations of the study. Given that the research assistant was present while the young person conducted the test, it may be argued that this does not reflect the way in which the test would be conducted in ‘real life’, or that it may not fully represent the participants’ views on the process. We only enrolled participants in the capital Gaborone, who may not necessarily accurately reflect the views of young people in Botswana. Furthermore, we recruited participants who came to the study clinic which likely biased our findings on the acceptability of self-testing because individuals who present to health facilities for SRH services may differ from those in the community who have not sought out such services. Participants who come to the clinic may have already had an interest in their sexual reproductive health, as evidenced by the fact that most of the young people already knew their HIV status. These individuals may be more accepting of self-testing because they are already engaged in healthcare-seeking behaviours related to their sexual reproductive health. Their views may therefore not be representative of those in the community who have never tested for HIV, particularly if they face barriers to accessing traditional healthcare services. These individuals may have different beliefs and concerns about self-testing that could impact the acceptability in the broader community. At the same time, the interest in testing for STIs could also mean dual-testing has the potential to promote re-testing in this high-risk population. Further research will be needed to explore the acceptability of self-testing among young people who have never tested for HIV, and reside in more remote parts of the country, as well as the potential for dual self-testing for HIV and STIs coupled with mobile health applications for remote counselling.

## Conclusion

Self-testing for HIV and STIs offers an opportunity to empower young people to become active agents in their own sexual health. It must however be linked with good quality counselling and effective mechanisms for linkage to care to ensure that young people are adequately supported throughout the process of self-testing.

## Supporting information

S1 Checklist Additional information regarding the ethical, cultural, and scientific considerations specific to inclusivity in global research. (DOCX)

S1 File PLOS ONE clinical studies checklist. (DOCX)

S2 File STROBE statement. (DOCX)
