# Correction: Youth and healthcare workers’ perspectives on the feasibility and acceptability of self-testing for HIV, Hepatitis and Syphilis among young people: Qualitative findings from a pilot study in Gaborone, Botswana

**DOI:** 10.1371/journal.pone.0295779

**Published:** 2023-12-07

**Authors:** Nambusi Kyegombe, Gbolahan Ajibola, Maureen Sakoi-Mosetlhi, Tsholofelo Rebatenne, Motswedi Anderson, Simani Gaseitsiwe, Joseph Makhema, Una Ngwenya, Sikhulile Moyo, Odile Sauzet, Lucy Mupfumi

The authors’ names are written incorrectly. The correct names are: Nambusi Kyegombe, Gbolahan Ajibola, Maureen Sakoi-Mosetlhi, Tsholofelo Rebatenne, Motswedi Anderson, Simani Gaseitsiwe, Joseph Makhema, Una Ngwenya, Sikhulile Moyo, Odile Sauzet, Lucy Mupfumi. The correct citation is: Kyegombe N, Ajibola G, Sakoi-Mosetlhi M, Rebatenne T, Anderson M, Gaseitsiwe S, et al. (2023) Youth and healthcare workers’ perspectives on the feasibility and acceptability of self-testing for HIV, Hepatitis and Syphilis among young people: Qualitative findings from a pilot study in Gaborone, Botswana. PloS ONE 18(7): e0288971. https://doi.org/10.1371/journal.pone.0288971
